# SNV/indel hypermutator phenotype in biallelic *RAD51C* variant - Fanconi anemia

**DOI:** 10.21203/rs.3.rs-2628288/v1

**Published:** 2023-03-02

**Authors:** Roni Zemet, Haowei Du, Tomasz Gambin, James R Lupski, Pengfei Liu, Paweł Stankiewicz

**Affiliations:** 1) Department of Molecular and Human Genetics, Baylor College of Medicine, Houston, TX; 2) Institute of Computer Science, Warsaw University of Technology, Warsaw, Poland; 3) Baylor Genetics, Houston, TX

**Keywords:** *de novo* variants, genome sequencing, hypermutation, multiple congenital anomalies, prenatal exome sequencing, perizygotic somatic mosaicism

## Abstract

We previously reported a fetus with Fanconi anemia (FA), complementation group O due to compound heterozygous variants involving *RAD51C*. Interestingly, the trio exome sequencing analysis also detected eight apparent *de novo* mosaic variants with variant allele fraction (VAF) ranging between 11.5%−37%. Here, using whole genome sequencing and a ‘home-brew’ variant filtering pipeline and DeepMosaic module, we investigated the number and signature of *de novo* heterozygous and mosaic variants and the rare phenomenon of hypermutation. Eight-hundred-thirty apparent dnSNVs and 21 *de novo* indels had VAFs below 37.41% and were considered postzygotic somatic mosaic variants. The VAFs showed a bimodal distribution, with one component with an average VAF of 25% (range: 18.7–37.41%) (n=446), representing potential postzygotic first mitotic events, and the other component with an average VAF of 12.5% (range: 9.55–18.69%) (n=384), describing potential second mitotic events. No increased rate of CNV formation was observed. The mutational pattern analysis for somatic single base substitution showed SBS40, SBS5, and SBS3 as the top recognized signatures. SBS3 is a known signature associated with homologous recombination-based DNA damage repair error. Our data demonstrate that biallelic *RAD51C* variants show evidence for defective genomic DNA damage repair and thereby result in a hypermutator phenotype with the accumulation of postzygotic *de novo* mutations, at least in the prenatal period. This ‘genome hypermutator phenomenon’ might contribute to the observed hematological manifestations and the predisposition to tumors in patients with FA, and pregnancy loss in general. We propose that other FA groups should be investigated for genome-wide *de novo* variants.

## Introduction

Fanconi anemia (FA) is a rare chromosomal instability syndrome that affects one in every 136,000 births (Mamrak et al. 2017). FA is a genetically and phenotypically heterogeneous disorder, resulting from perturbations in genes involved in DNA repair and cell cycle regulation. When the cell cannot sufficiently repair its genetic information, and genomic integrity is compromised, various clinical manifestations, such as congenital malformations, early progressive bone marrow failure, and predisposition to hematologic malignancies and solid tumors occur (Auerbach 2009; Kolinjivadi et al. 2020; Mehta and Ebens 2021; Moldovan and D’Andrea 2009; Rageul and Kim 2020). Physical abnormalities, present in approximately 75% of affected individuals with FA, include one or more of the following: endocrine dysfunctions leading to short stature, abnormal skin pigmentation, skeletal deformities of the thumb and forearm, microcephaly, and ophthalmic and genitourinary tract anomalies (Auerbach 2009; Ceccaldi et al. 2016; Kee and D’Andrea 2012; Mehta and Ebens 2021). In addition, FA patients are predisposed to various cancers, including acute myeloid leukemia (AML) and squamous cell carcinoma of the head and neck (Kutler et al. 2016; Kutler et al. 2003).

To date, variants in more than 20 genes *(FANCA* to *FANCW)* have been found to cause FA as an autosomal recessive (AR) trait, in *FANCB* exhibits X-linked (XL) inheritance, and in FANCR/RAD51, which have been associated with an autosomal dominant (AD) rare disease trait (Ameziane et al. 2015; Badra Fajardo et al. 2022; Moreno et al. 2021; Wang et al. 2015). All proteins form an integrated protein network, known as the FA/BRCA repair pathway (Ceccaldi et al. 2016). The network is active during DNA replication when converging replisomes encounter damage that covalently binds the two DNA strands (interstrand crosslinking; ICL). Sequential recruitment of FA proteins and associated partners onto chromatin unhooks the ICL and coordinates the repair through homologous monoubiquitination and recombination (Badra Fajardo et al. 2022; Moreno et al. 2021; Nalepa and Clapp 2018). Emerging evidence suggests that independent of ICL and homologous recombination (HR) repair, FA proteins also regulate cell-cycle checkpoints and/or promote replication fork remodeling in response to replication stress, redefining the FA pathway as a cardinal mechanism to preserve genome integrity throughout the entire replication process (Badra Fajardo et al. 2022).

Alterations in FA genes have been found to incite genomic instability and contribute to tumorigenesis (Badra Fajardo et al. 2022). Accordingly, cells from FA patients are hypersensitive to ICL-inducing agents such as diepoxybutane (DEB) and mitomycin C (MMC), which cause high levels of chromosomal aberrations, including chromosomal breaks and quadriradial formation. In addition to identifying pathogenic variants in the FA genes, this unique and characteristic cellular phenotype is still employed for the objective clinical laboratory diagnostics of FA patients using a DEB and MMC-induced chromosome breakage test of lymphocytes (Rageul and Kim 2020).

FA proteins are classified into three general groups based on the biochemical activities and functions in ICL repair: FA core complex, which recognizes the damage and ubiquitylates the heterodimer (FANCI-FANCD2), ID2 heterodimeric complex formation, which recruits repair factors, such as nucleases (FANCP and FANCQ), trans-lesion synthesis polymerases (FANCV and polymerase ζ), and HR factors (FANCD1, FANCJ, FANCN, FANCO, FANCR, FANCS, and FANCU) (Duxin and Walter 2015; Kolinjivadi et al. 2020; Milletti et al. 2020). Several FA proteins from the third group participate in HR, a double-strand break (DSB) repair pathway, which is active during the S and G2 cell-cycle phases; HR utilizes extensive sequence homology from a donor template for error-free repair of broken DNA ends. Furthermore, monoallelic mutations in the FA genes within the HR pathway, including *RAD51C* (FANCO), *BRCA2* (FANCD1), *BRCA1* (FANCS), *BRIP1* (FANCJ), or *PALB2* (FANCN), have been associated with familial predisposition to breast, ovarian, and pancreatic cancers (Ceccaldi et al. 2016; D’Andrea 2010; Kottemann and Smogorzewska 2013; Rageul and Kim 2020). RAD51C has also been shown to be involved in intra-S-phase checkpoint regulation through CHK2 activation in response to DNA damage (Somyajit et al. 2012; Vaz et al. 2010). *Rad51c* deficient mice show early embryonic lethality (Kuznetsov et al. 2009), whereas mice carrying a null and a hypomorphic allele show infertility with partial penetrance (Kuznetsov et al. 2007).

Previous whole genome sequencing (WGS) and exome sequencing (ES) studies have provided insights into the scale of *de novo* variants in the normotypical population and as a cause of genetic diseases (Acuna-Hidalgo et al. 2016; Veltman and Brunner 2012). The mutation rate of single nucleotide variants (SNVs) has been estimated at 1.0–1.8×10^−8^ variants per base per generation, giving rise to 60–70 *de novo* variants per genome, with one to two affecting the coding sequence (Campbell and Eichler 2013; Goldmann et al. 2016; Kong et al. 2012; Roach et al. 2010). It is estimated that locus-specific spontaneous mutation rates for copy-number variants (CNVs) are approximately hundreds or thousands fold higher than that of *de novo* SNVs (dnSNVs), *i.e*. ~ 10^−6^ to 10^−4^ per generation, resulting in 0–1 *de novo* CNV per genome (Lupski 2007; Turner et al. 2008).

Interestingly, Liu et al. (Liu et al. 2017) and Du et al. (Du et al. 2022) described a novel type of constitutional genome instability with an unusually large number of *de novo* mutations (DNMs) for multiple *de novo* CNVs (MdnCNV) occurring during perizygotic mutagenesis; this MdnCNV phenomenon showed evidence for regional SNV hypermutagenesis in a 4 Mb ‘window’ surrounding the CNV breakpoint junctions consistent with replicative recombination repair involving an error prone polymerase (Kaplanis et al. 2022; Liu et al. 2017). Most MdnCNVs were arranged as large tandem duplications (~1 Mb in size) with microhomology and microhomeology at the breakpoints and *dn*SNVs in their vicinity. Genetic marker studies revealed the M*dn*CNV arose in a perizygotic time interval of organismal development, thus affecting all cells of the human body.

In a more recent study, germline hypermutation of dnSNVs was identified in genome-wide studies from 12 individuals out of 21,879 families with rare genetic diseases. The number of dnSNVs for each individual with hypermutation ranged from 110 to 425, correlating to a 1.7–6.5 fold increase compared with the median number of dnSNVs in the general population. Two of these individuals also had a significantly increased number of *de novo* insertion/deletions (indels) (Kaplanis et al. 2022). Constitutional new variants have been considered to primarily arise from germline or zygotic events; however, more recent data suggest postzygotic new variants are an under-recognized source of *de novo* genomic variations (Acuna-Hidalgo et al. 2015; Rahbari et al. 2016). Postzygotic events contribute to the formation of mosaicism, and recent advances in genomic technologies have enhanced our ability to detect and characterize low-level mosaicism (Contini et al. 2015; Doan et al. 2021; Lannoy and Hermans 2020; Uchiyama et al. 2016).

In 2018, we reported a newborn female with an expanded phenotype of Fanconi anemia, complementation group O (FANCO) (Jacquinet et al. 2018). She was diagnosed prenatally with several congenital anomalies: bilateral ventriculomegaly, absence or fenestration of the septum pellucidum and fusion of the fornices anteriorly, thick and echogenic corpus callosum, cleft lip and palate, overlapping fingers, heart anomalies, symmetric fetal growth restriction, and suspected ambiguous genitalia. Of note, the family history was significant for breast cancer in the paternal grandmother and great-grandmother. Chromosomal microarray analysis using Affymetrix CytoScan HD SNP array performed on DNA from amniotic fluid was normal. Prenatal trio ES on DNA isolated from cultured amniocytes revealed inherited compound heterozygous variant alleles in *RAD51C*: (NC_00017.10(NM_058216.2)): c.935G>A (p.Arg312Gln) and c.571+5G>A that were interpreted as likely pathogenic. In addition, trio ES analysis detected eight apparent *de novo* mosaic variants in the fetus with variant allele fractions (VAF) ranging between 11.5% and 37% (Jacquinet et al. 2018). Chromosome breakage studies confirmed the diagnosis of FANCO, while the cleft lip and palate and the lobar holoprosencephaly were considered an expansion of the phenotypic spectrum of FANCO. The child died soon after birth.

Here, we describe the results of subsequent trio WGS studies in the family, which revealed SNV hypermutagenesis as evidenced by a large number of apparent dnSNVs and indels.

## Materials and Methods

### Genomic sequencing

Fetal DNA was extracted from amniotic fluid and parental DNA was extracted from peripheral blood. The prenatal trio ES and WGS were performed on the Illumina HiSeq platform following standard protocols as previously described (Liu et al. 2017; Normand et al. 2018; Yang et al. 2013). The total mean autosomal sequencing read depth-of-coverage in WGS ranged between 42–58x per sample (**Supplementary Table 1**).

### Selection criteria for candidate *de novo* variants

A custom bioinformatics script was utilized to detect and filter apparent *de novo* SNVs or small indels in the trio WGS data (Gambin et al. 2020). We analyzed the VCF file to select variants for which the proband was found to be heterozygous by calculating the VAFs. We have previously shown that more than 95% of apparent *de novo* autosomal SNVs and X-linked SNVs in females in ES analyses have VAF ranging between 37.41–62.6% (Cao et al. 2019). We have now used more stringent criteria to eliminate genotype calls erroneously classified as heterozygous and removed variants with VAF above 70%, variants with a total depth of coverage below 20x in any sample from the trio, and DNMs overlapping known segmental duplications, centromeres, or *Alu* repetitive elements. We have included variants (SNVs or indels) with ≥2 alternative reads in the proband and absent in both parents. To further reduce the number of false positives and technical artifacts, we have removed all variants present in gnomAD v3.1 database [https://gnomad.broadinstitute.org]. For each selected variant, we have retrieved pileup information from the proband and parental BAM files that enabled obtaining more precise data on read depth and VAF in these samples. The analyses of the pileup data were performed using Samtools version 1.13 (Danecek et al. 2021). We used hard filtering based on https://gatk.broadinstitute.org/hc/en-us/articles/360035890471-Hard-filtering-germline-short-variants [RMSMappingQuality > 30 (for SNPs), ReadPosRankSum >−8 (SNPs) or ReadPosRankSum >−20 (InDels), and FisherStrand < 60 (SNPs) or FisherStrand < 200 (InDels), QualByDepth > 2]. All *de novo* SNVs and indels were manually curated via Integrative Genomics Viewer (IGV, v2.3) software (Robinson et al. 2011; Thorvaldsdottir et al. 2013) with the previously described criteria (Du et al. 2022).

To validate the customized variant filtering, we have used the recently published prediction module DeepMosaic, which combines an image-based visualization for mosaic SNVs with a convolutional neural network-based classification for mosaic variants detection. This pipeline has an increased sensitivity (using HaplotypeCaller with ploidy=50) and fully automated filtration mechanism (Yang et al. 2023). The *de novo* variants observed in the trio WGS of the FA patient was compared to the *de novo* variants in the control trio WGS using both pipelines.

### De novo substitution mutational signature pattern analysis

The R package MutationalPatterns (Manders et al. 2022) was used for *de novo* substitution mutational signature analysis. The tri-nucleotide and pan-nucleotide mutational contexts were extracted and visualized with ‘mut_matrix’ and ‘plot_96_profile’ functions from the MutationalPattern R package. Known signatures from COSMIC (v3.2) were refitted using the ‘backwards’ method. The method starts by achieving an optimal reconstruction via ‘fit_to_signatures.’ The signature with the lowest contribution is then removed and refitting is repeated iteratively. Each time the cosine similarity between the original and reconstructed profile is calculated using refitting with ‘backwards’ method.

### Refitting bimodal VAF distribution

To infer the timing within the life cycle of the *de novo* variants event, we have evaluated their VAF distribution (density plot). We have used a custom R code to identify the boundaries corresponding to the first, second, and third cell division, looking for the points of intersection between different distributions. To this end, we have sampled 10,000 values from theoretical densities of binomial distributions of VAFs corresponding to each cell division using the cbinom R package (https://cran.r-project.org/web/packages/cbinom/cbinom.pdf). Next, we have estimated the boundaries between the consecutive pairs of distributions by identifying the positions closest to the intersection points.

### CNV calling and visualization

CNVs were called using Illumina Dragen Bio-IT Platform (v3.4.15). The read depth was calculated with mosdepth (v 0.3.4) (Pedersen and Quinlan 2018) and visualized with the in-house visualization tool VizCNV (https://github.com/BCM-Lupskilab/VizCNV) that allows for normalized read depth plotting of the proband and both parents to help with manual inspection of potential *de novo* CNVs larger than 3 kb.

## Results

### De novo variants identification in ES and WGS data

Computational CNV analyses followed by manual read-depth visualization did not reveal any increased rate of CNV formation (**Supplementary Figure 1**). Using the customized variant filtering pipeline, genome-wide analyses of the trio WGS data for the FA patient revealed 45 *de novo* variants with VAFs ranging between 37.41% and 62.6%, considered as germline variant alleles. In the control trio WGS, 34 *de novo* variants had the same germline VAF range.

For VAFs below 37.41%, considered as likely postzygotic somatic mosaic events, after removal of the false-positive variants during IGV inspection, an unexpectedly high number of apparent dnSNVs (n=830) and indels (n=21) were detected, compared to only 11 dnSNVs and 3 indels in the control case (Table 1a). Using the DeepMosaic module, analyses of trio WGS data from the FA patient identified 44 non-mosaic heterozygous variants, and 850 dnSNVs and 25 indels with VAF below 37.41%, compared to 34 heterozygous variants and 38 dnSNVs and 8 indels with VAF below 37.4% in the control sample (Table 1b) (**Supplementary Table 2**).

Re-analysis of prenatal trio ES data confirmed eight apparent *de novo* mosaic variants with VAF ranging between 11.5% and 37% and revealed an additional 15 apparent *de novo* mosaic variants with VAFs ranging between 9% and 37% (**Supplementary Table 3**). The novel variants are mainly located in non-coding regions, close to the exon boundaries. Although the number of *de novo* mosaic variants detected through the exome analysis is not high, the depth-of-coverage is sufficient to consider the VAFs of the mosaic variants as solid.

### De novo variant allele frequency distribution

The VAF pattern of apparent dnSNVs (Figure 1) suggests their bimodal distribution, with one component with an average VAF of 25% (ranging between 18.7–37.41%) (n=446), representing potential postzygotic first mitotic events, and the other with an average VAF of 12.5% (ranging between 9.55–18.69%) (n=384), representing potential second mitotic events.

### Mutational signature of the *de novo* substitutions

Genome-wide distribution of the somatic *de novo* substitutions shows clusters (genomic distance <50 bp) spreading across multiple chromosomes (Figure 2a). Clustered variants are likely generated by the same mutagenic events and may provide insights into the somatic mutational processes (Alexandrov et al. 2020; Supek and Lehner 2017). Distinct patterns were observed after reviewing the pileup of *de novo* variants in IGV. We have classified the *de novo* substitutions into four categories: single-base substitution (SBS, n=799), tandem dinucleotide or duplet-base substitution (DBS, n=9), multiple (n>1) single-base substitution within 50 bp in distance or clustered-base substitution (CBS, n=13), and biallelic base substitution (BBS, n=3), in which two substitutions are present at the same location (Figure 2 b–e). Of note, a higher number of SBS, DBS, and CBS were observed in the FA patient compared to the control case (Table 2). The CBS does not seem to represent the kataegis phenomenon, where a much large number of base pair mutations occur in a small region with enrichment of transition substitutional pattern (C>T or G>A) (54). Most of the CBS observed in the FA patient were in two base pair variants in a distance of 1 bp and enriched for transversion over the transition (Ti:Tv = 0.04). We compared SBS to the COSMIC context to infer potential somatic mutational processes. The mutational pattern analyses of the SBS revealed enrichment of C>G, C>A, and C>T variants (Figure 3). The mutational pattern analysis for somatic SBS shows SBS40, SBS5, and SBS3 as the top signatures (Figure 4). The SBS40 is a flat signature similar to SBS5, of which the underlying etiology is uncertain. SBS3 is a known signature associated with HR-based DNA damage repair error, often due to *BRCA1* or *BRCA2* inactivation (Nik-Zainal et al. 2012).

## Discussion

RAD51 is a RecA-like DNA recombinase that initiates HR upon DNA damage by replacing Replication protein A (RPA) and catalyzing strand transfer between the broken sequence and its undamaged homolog (Boni et al. 2022). RAD51C is a member of the RAD51 family, RAD51B, RAD51C, RAD51D, XRCC2, and XRCC3, required for efficient DNA double-strand break repair by HR (Chun et al. 2013). Furthermore, RAD51 paralogs have been shown also to play cardinal roles in protecting the replicative fork during DNA synthesis (Somyajit K 2015), avoiding unrestrained fork progression, and promoting efficient restart (Berti et al. 2020). Depletion of RAD51 paralog genes in human cell lines has been associated with impaired HR, reduced genome stability, increased DSBs, and growth defects (Boni et al. 2022). The collection of replication stress-associated DNA lesions leads to genomic instability and may predispose to cancerogenesis, also in the heterozygous state, *e.g*., cancers arising mainly in the breast and ovary (Badra Fajardo et al. 2022; Boni et al. 2022; Kottemann and Smogorzewska 2013).

The first pathogenic variant in *RAD51C* associated with a human disorder was described in three siblings from a consanguineous Pakistani family with clinical features suggestive of FA (Vaz et al. 2010). Functional analyses of the homozygous missense mutation (NM_058216.2:c.773G>A (p.Arg258His)) demonstrated an increase in G2 arrest in response to MMC in patient cultured lymphocytes compared to controls, a decrease in RAD51C focus formation in response to MMC in patient fibroblasts, a modest radiosensitivity, and an increased sensitivity to the topoisomerase I inhibitor, camptothecin (Somyajit et al. 2012; Vaz et al. 2010). Since the report of this family, only the presented individual with FANCO has been reported in the literature, thus the phenotypic spectrum associated with *RAD51C* mutations remains obscure.

The ‘mutator phenotype’ was first described in *Drosophila* and in bacteria (Liu et al. 2017; Miyake 1960; Plough 1941). In the last two decades, it has been appreciated also in cancers (Nicolaides et al. 1998). The variants have been found to be usually driven by an intrinsic source, such as a defective mismatch repair gene (Loeb 2001). Therefore, the mutations are expected to develop over time, and accumulate in a somatic mosaic state (Kilpivaara and Aaltonen 2013). In contrast, the non-cancer constitutional CNV mutator phenotype described by Liu et al. (Liu et al. 2017) and most recently further expanded by Du et al. (Du et al. 2022) seems to be restricted to a specific time-interval in early embryonic development and no longer operating after early postzygotic division, as evidenced by the lack of mosaicism for any dfoCNVs over time. Nevertheless, several features of the constitutional MdhCNV phenomenon suggested that these mutations arise due to a faulty replicative repair process, resembling chromosomal instability in various cancers. The presence of complex genomic rearrangements, microhomology and microhomeology observed at breakpoint junctions, templated insertion at the breakpoints, and dŜNVs in the proximity (~1–4 Mb) to breakpoint junctions are characteristics of the fork stalling and template switching (FoSTeS)/microhomology-mediated break-induced replication (MMBIR) mechanisms (Bahrambeigi et al. 2019; Beck et al. 2019; Carvalho and Lupski 2016; Carvalho et al. 2013; Lee et al. 2007).

Recently, Kaplanis et al. proposed three potential sources of germline hypermutation identified in 12 individuals: (i) defects in paternal DNA-repair genes (*XPC*, involved in the early stages of the NER pathway, and *MPG*, involved in recognition of base lesions, including alkylated and deaminated purines, and initiation of the base-excision repair pathway), (ii) paternal exposure to chemotherapeutics, and (iii) postzygotic mutational factors (Kaplanis et al. 2022). One patient with postzygotic mutational factors had several blood-related clinical phenotypes, including myelodysplasia, and the observations were assumed to be related to clonal hematopoiesis, leading to a large number of somatic mutations in the child’s blood. The hematopoietic presentation is shared with FA patients, although the DNA repair process differs. There were no similar blood-related phenotypes in the second patient with postzygotic mutational factors, although the child was only one year old at recruitment.

The distribution pattern of the VAF for the mosaic dnSNVs showed a bimodal distribution, with most of the VAFs oscillating around 25% and 12.5%, indicating these variants likely arose during the first and second mitotic division (Ju et al. 2017; Oron and Ivanova 2012). Relatively low read coverage in WGS data and lack of postnatal proband DNA precluded us from analyzing lower-level mosaic variants potentially corresponding to subsequent mitotic divisions. More systematic studies of ultra-high coverage may better inform on a complex interplay of mechanisms surveilling early postzygotic genome stability, *e.g*. protective maternal cytoplasmatic proteins (Schulz and Harrison 2019) and more efficient DNA-repair leading to a reduced mutation rate proposed to be established from the 4-cell stage onwards (Coorens et al. 2021; Park et al. 2021).

Distinctive mutational patterns, termed ‘mutational signatures,’ are associated with different mutational mechanisms (Alexandrov et al. 2013). There are more than 100 somatic mutational signatures defined across various cancers, of which some have been attributed to endogenous mutagenic processes (Phillips 2018). Most germline mutations can be accounted for by two signatures, SBS1 and SBS5. Both signatures are ubiquitous among normal and cancer cells (Moore et al. 2021). The mutational signatures identified in our patient exhibited known single base substitution signatures (SBS1, SBS5), including one associated with HR-based DNA damage repair error (SBS3), consistent with *RAD51C* role in HR-mediated repair of ICL and double-strand DNA breaks (Somyajit et al. 2012; Vaz et al. 2010). In Kaplanis et al., the two individuals with postzygotic mutational factors shared a large contribution from SBS1 (Kaplanis et al. 2022).

We propose that biallelic *RAD51C* mutations and defects in DNA damage response result in a ‘hypermutator phenotype’ with the accumulation of postzygotic *de novo* mutations. Recently reported mutational spectrum of squamous cell carcinoma from individuals with FA showed a collection of somatic SV, with exposure to tobacco and alcohol mutagens overwhelming the ability of a defective DNA repair pathway (Webster et al. 2022). The absence of major SV in our patient could be due to the more stringent selection pressure that occurs during embryonic development compared to selection for cancer cells. We cannot exclude the possibility that somatic mosaic SVs could contribute to the clinical phenotype of this patient later in life, e.g. AML or squamous cell carcinoma of the head and neck.

Heterozygous carriers of variants involving the FA genes from the HR pathway, including *RAD51C*, have been associated with familial predisposition to breast-ovarian cancer (D’Andrea 2010; Rageul and Kim 2020). Germline mutations in *RAD51C* have been identified in about 1% of hereditary breast and ovarian cancer families (the lifetime risk of ovarian cancer is approximately 9%) and in families with a history of breast cancer alone (Sopik et al. 2015). Importantly, in the present case, the family history was significant for breast cancer in female family members of the proband’s father with the heterozygous splicing variant c.571+5G>A in *RAD51C*. Interestingly, the same variant was identified in a Chinese individual among 273 BRCA1/2-negative familial breast cancer cases (Pang et al. 2011). Segregation analysis of the paternally inherited splicing variant (c.571+5G>A) was recommended to better counsel the family about the increased risk for breast and ovarian cancer in female carriers. Similar segregation should also be considered for the maternally inherited missense variant, although it was not previously described in familial breast cancer cases. Of note, reproductive options, such as prenatal diagnosis and preimplantation genetic testing, were discussed with the parents due to 25% risk of recurrence of FA.

Finally, we propose that in addition to chromosome aneuploidies, common during early human embryogenesis (Cavazza et al. 2021; Vanneste et al. 2009), the hypermutation phenomenon, such as observed in our patient with FA, might represent the underrecognized mechanism responsible for early pregnancy losses.

## Conclusions

Our data demonstrate that biallelic *RAD51C* variants affecting DNA damage repair process result in an SNV hypermutator phenotype, leading to the accumulation of postzygotic *de novo* variants, at least in the prenatal period. This phenomenon might contribute to the hematological manifestations and the predisposition to cancer in patients with FA. Investigating this phenomenon may enhance our knowledge about the phenotype of FA and cancer biology. We propose that additional FA groups be analyzed genome-wide for *de novo* variants and better genotype-phenotype correlations. As most DNA-repair disorders, including FA, are recessive disorders, we suggest that genetic causes of hypermutation are more likely to be found at higher frequencies in populations with increased consanguinity rates. Importantly, hypermutation phenotype may be an under-ascertained factor responsible for early pregnancy losses.

## Figures and Tables

**Fig. 1.** *De novo* variant allele frequency distribution VAF density plots in the FA proband (red histogram) with two components of the bimodal distribution (red curve line) compared with the control subject (blue histogram and line) generated using the home-brew pipelines: (i) with manual IGV curation step (A), and (ii) with increased sensitivity (ploidy = 50 in GATK HC) and automated filtration using DeepMosaic (B). The lines denote the potential postzygotic mutational events (VAF <37.4%) and the potential germline cell events (VAF 37.41–62.6%).

**Fig. 2.** Genome-wide distribution of *de novo* substitution and examples of *de novo* substitution pattern in the FA patient a. Rainfall plot of *de novo* substitutions. b-e. Integrative Genomics Viewer (IGV) visualization of the proband (top) and parents (bottom) short read sequencing data at four loci, illustrating four different substitution patterns: b) Single-base substitution (SBS), c) double-base substitution (DBS), d) clustered-base substitution (CBS), e) biallelic-base substitution (BBS)

**Fig. 3.** Mutational pattern of *de novo* single base substitution. a) 96 tri-nucleotide profile b) Pan-nucleotide river plot with the extended context (2 bp)

**Figure 4.** Mutational single base substitution signature. a) The top 20 known single base substitution (SBS) signatures ranked by the cosine similarity (from top to bottom). b) The absolute contribution of the fitted SBS signatures.

**Table 1.** Distribution of VAFs among the potential *de novo* heterozygous and mosaic SNVs/InDels

a. Customized variant filtering pipeline						
VAF range	Fanconi SNVs	Fanconi InDels	Fanconi All	Control SNVs	Control InDels	Control All
**9.55% - 18.69%**	384	8	392	8	3	11
**18.7% - 37.41%**	446	13	459	3	0	3
**37.41 – 62.59%**	45	5	50	34	6	40
b. DeepMosaic filtering pipeline						
VAF range	Fanconi SNVs	Fanconi InDels	Fanconi All	Control SNVs	Control InDels	Control All
**9.55% - 18.69%**	413	9	422	35	7	42
**18.7% - 37.4%**	437	16	453	3	1	4
**37.41 – 62.59%**	44	5	49	34	6	40

SNV, single nucleotide variant; VAF, variant allele frequency

**Table 2.** Mutational signature analysis for *de novo* substitutions: Breakdown of the germline and somatic variants in the FA proband and control

		Fanconi Anemia patient				Control			
	Mutation type	Transition (Ti)	Transversion (Tv)	Total	Ti/Tv	Transition (Ti)	Transversion (Tv)	Total	Ti/Tv
**Germline**	SBS	27	40	67	0.68	22	13	35	1.69
	DBS	0	0	0	NA	0	0	0	NA
	CBS	2	0	2	NA	0	0	0	NA
	BBS	NA	NA	0	NA	NA	NA	0	NA
	Total	29	40	69	0.73	22	13	35	1.69
**Somatic**	SBS	278	521	799	0.53	4	3	7	1.33
	DBS	6	12	18	0.50	0	0	0	NA
	CBS	1	23	24	0.04	1	2	3	0.50
	BBS	NA	NA	3	NA	NA	NA	0	NA
	Total	285	556	844	0.51	5	5	10	1.00

BBS, Biallelic base substitution; CBS, Clustered-base substitution; DBS, Doublet-base substitution; SBS, Single-base substitution; NA, not applicable

## Data Availability

The dataset supporting the conclusions of this article is included within the article (and its additional files).
